# Flame spray pyrolyzed carbon-encapsulated Au/Fe_3_O_4_ nanoaggregates enabled efficient photothermal therapy and magnetic hyperthermia of esophageal cancer cells

**DOI:** 10.3389/fbioe.2024.1400765

**Published:** 2024-05-28

**Authors:** Zida Wang, Gongzhe Liu, Jiangping Zhou, Xiaogang Zhao, Jie Cai

**Affiliations:** ^1^ Department of Emergency, Shanghai Pulmonary Hospital, School of Medicine, Tongji University, Shanghai, China; ^2^ Department of Cardiothoracic Surgery, People’s Hospital Affiliated to Shandong First Medical University, Jinan, China; ^3^ Department of Anesthesiology, Shanghai Pulmonary Hospital, School of Medicine, Tongji University, Shanghai, China; ^4^ Department of Thoracic Surgery, Shanghai Pulmonary Hospital, School of Medicine, Tongji University, Shanghai, China

**Keywords:** magneto-plasmonic nanoparticles, carbon encapsulation, core–shell, photothermal therapy, magnetic hyperthermia, magnetic resonance imaging

## Abstract

Multifunctional magneto-plasmonic nanoparticles with magnetic hyperthermia and photothermal therapy could kill cancer cells efficiently. Herein, carbon-encapsulated Au/Fe_3_O_4_ (Au/Fe_3_O_4_@C) was fabricated using an enclosed flame spray pyrolysis. The nanostructures, including an Fe_3_O_4_ core (51.9–55.2 nm) with a decreasing carbon shell thickness and an Au core (4.68–8.75 nm) coated with 2–4 graphite layers, were tailored by tuning the C_2_H_4_ content in the reacting gas mixture. Saturation magnetization (33.7–48.2 emu/g) and optical absorption were determined. The carbon shell facilitated the dispersion of Au/Fe_3_O_4_ and restrained their laser-induced and magnetic field-induced coalescence and growth. Au/Fe_3_O_4_@C exhibited excellent magnetic resonance imaging capability (91.4 mM^−1^ s^−1^) and photothermal performance (65.4°C for 0.8 mg/mL Au/Fe_3_O_4_@C at a power density of 1.0 W/cm^2^ after 300 s near-IR laser irradiation (808 nm)). Moreover, the combined application of photothermal and magnetic-heating properties reduced the required intensity of both laser and magnetic field compared to the intensity of separate situations. Our work provides a unique, intriguing approach to preparing multicomponent core/shell nanoaggregates that are promising candidates for esophageal cancer cell therapy.

## 1 Introduction

Cancer is a severe public health problem that seriously threatens human health. According to GLOBOCAN 2020 data, approximately 2,000,000 cases of cancer were reported, and the case-death ratio was more than 50% worldwide in 2020 (Alifu et al., 2022). Although we have made great success in the field of anticancer research, commonly applied approaches for the treatment of cancers are still restricted due to multiple reasons, including inevitable adverse damage to normal tissues, resistance to existing drugs, and scarcity of specific therapy (Shrestha et al., 2018). Therefore, it is necessary to find other available treatment methods, especially those that can achieve a centralized treatment, to achieve the therapeutic effect and adaptability for patients. In recent years, hyperthermia has attracted great attention via heating cancer cells or tissues to death. There is an obvious breaking point in the response of cells to heat, according to the *in vitro* experiments (Huang et al., 2022). The slope of the survival curve is shallower at temperatures below the breaking point because the heat shock protein is expressed to protect cells from further damage, thus inducing heat resistance. However, tumor blood vessels may collapse above the breakpoint temperature, thus capturing the applied heat and leading to cell necrosis or apoptosis. For every 1°C increase in temperature, the cell death rate will double. Currently, 43°C has been set as the basic effective temperature of hyperthermia (Shi et al., 2024).

A magneto-caloric process induced by the heating of magnetic nanoparticles (NPs) has triggered a feasible nanoplatform for the specific therapy of cancer cells (Beik et al., 2019; Mirrahimi et al., 2020; Roquero et al., 2021). By regulating the applied magnetic field, these magnetic NPs play a major role in transforming magnetic energy into heat energy. After magnetic NPs were injected into tumors with the assistance of an applied alternating field, the localized temperature of tumors increased rapidly due to the magnetic loss. Because tumor cells are more sensitive to temperature rise than normal cells, the local temperature of tumor tissue will rise sharply to destroy tumor cells and achieve therapeutic effects (Jeong et al., 2021; Niraula et al., 2024). Compared with other physical stimuli widely used in medicine, remote-controlled magnetic heat with nanoscale spatial resolution can penetrate tissues without restricted depth and weakened intensity. The localized temperatures of tissues can also be finely regulated (Rajan and Sahu, 2021). Among various types of magnetic nanomaterials, iron oxide NPs have presented multiple advantages, including strong magnetism response, good biological compatibility, and inexpensive production costs (Espinosa et al., 2021; Xu et al., 2023). These NPs could be guided to the required position by applying a magnetic field and tracked using magnetic resonance imaging (MRI). Iron oxide NPs, which are easily customizable with more functions through surface functionalization and component integration, display great potential in the treatment and diagnosis of cancers.

Photothermal therapy is also considered a promising tumor treatment method because of its low invasiveness and high tumor targeting (Eyvazzadeh et al., 2017; Ji et al., 2023). Photothermal therapy targets cells that are more susceptible to high temperatures (Ghaznavi et al., 2018; Farashahi et al., 2019). In the early stage of laser irradiation for tumor treatment, the heat generated by the external laser may damage the healthy tissues around the tumor area (Mirrahimi et al., 2018). Nowadays, near-infrared light (NIR) has been applied to the effective position with a photothermal agent. The aggregation of exogenous photothermal agents in tumor cells is greater than that in surrounding healthy tissue cells, which reduces the damage of photothermal therapy to adjacent normal tissues and gives photothermal therapy a good curative effect and high selectivity.

The ideal photothermal agent not only has high photothermal conversion efficiency but also aggregates in tumor cells. To further improve the effect of photothermal therapy, nanomaterials have been applied to overcome the limitations of photothermal therapy (such as high temperature or laser damage to normal cells) and the toxic and side effects on normal human tissues (Liu et al., 2020; Zhou et al., 2020). Photothermal nanomaterials such as metallic nanomaterials, semiconductor nanocrystals, and carbonaceous nanomaterials have been exploited (Wang Y. F. et al., 2021; Zhang L. X. et al., 2021; Du et al., 2022; Li et al., 2023; Liu et al., 2023; Lu et al., 2023). Among them, metal-based nanomaterials stand out in biomedicine because of their unique properties of electricity, magnetism, light, surface plasma resonance, stability, and easy modification (Li et al., 2019; Qi et al., 2020; Zhao et al., 2021; Fan et al., 2022).

Photothermal conversion agents have also been applied as contrast agents for biological imaging to conduct more accurate tumor thermal ablation, reduce the damage to surrounding normal tissues, and monitor the situation of tumor areas. Metal-based nanomaterials can achieve better tumor treatment effects by combining photothermal therapy, biological imaging, and other tumor treatment methods (Wang et al., 2018; Zhou et al., 2018; Luo et al., 2020; Zhang et al., 2020). For example, Au nanoparticles could be used as a photothermal conversion agent with good performance in photothermal therapy and as an effective contrast agent in biological imaging (Natanael et al., 2020; Zhang Y. et al., 2021; Wang et al., 2022). After the surface modification, the NPs could be loaded with drugs to treat tumor cells by laser-controlled release, significantly improving the efficiency of tumor treatment (Karunanidhi et al., 2021; Pinakidou et al., 2022). Above all, the unique advantages of metal-based nanomaterials provide a great impetus for the preparation and application of metal-based nanomaterials in the fields of biological imaging and photothermal therapy.

In this work, multifunctional carbon-encapsulated Au/Fe_3_O_4_ nanoaggregates (Au/Fe_3_O_4_@C NGs) with tailorable carbon shell thicknesses were synthesized using enclosed flame spray pyrolysis. The morphologies, elemental compositions, crystalline structures, optical absorption, and magnetic properties of Au/Fe_3_O_4_@C NGs were characterized. Au/Fe_3_O_4_@C NGs were applied as X-ray computerized tomography imaging contrast agents, photothermal agents, and magnetic hyperthermia agents. The hyperthermia of esophageal tumor cells was carried out to evaluate the therapy efficiency of Au/Fe_3_O_4_@C NGs. Moreover, the heating efficiency of Au/Fe_3_O_4_@C NGs under the combination of laser radiation and magnetic field was also investigated.

## 2 Materials and methods

### 2.1 Materials

Gold acetate (purity >99.5%), ferric acetate (purity >99.8%), acetonitrile (purity >99.5%), and 2-ethylhexanoic acid (purity >99.5%) were obtained from Sigma-Aldrich Co., Ltd., and used as received without further purification. Ethylene (C_2_H_4_, purity >99.9%) and oxygen (O_2_, purity >99.9%) were obtained from Nanjing Shangyuan Gas Factory. Other chemicals were of reagent grade from Aldrich.

### 2.2 Preparation of Au/Fe_3_O_4_@C NGs

Multicomponent Au/Fe_3_O_4_@C NGs were fabricated via enclosed flame spray pyrolysis. Typically, gold acetate with a concentration of 0.2 mol/L and ferric acetate with a concentration of 0.2 mol/L were dissolved into the solvents of acetonitrile and 2-ethylhexanoic acid (1:1 of volume) under constant stirring. This precursor solution of Au/Fe_3_O_4_ was injected into the reaction chamber with a feed speed of 5 sccm. O_2_ gas with a flow of 50 sccm was applied to sheathe this solution. The enclosed flame spray pyrolysis was ignited in a quartz glass tube (40 mm diameter), which was preheated via adding xylene for 5 min before the particle fabrication process. A premixed gas of C_2_H_4_/O_2_ (4.0 L/min with three specific ratios) was utilized as the carbon source. The as-prepared NGs were collected with the assistance of a glass fiber filter. The content of coating carbon in Au/Fe_3_O_4_@C NGs was regulated to the given values by changing the ratio of C_2_H_4_ in the C_2_H_4_/O_2_ gas mixture.

### 2.3 Characterization of Au/Fe_3_O_4_@C NGs

Transmission electron microscopy (TEM) was carried out via a Hitachi H-7100 instrument. X-ray diffraction (XRD) was carried out via a D8 Advance X-ray diffractometer (Bruker, Germany). Energy-dispersive X-ray spectroscopic (EDS) line profiles were recorded via a scanning electron microscope (Hitachi SU8700). X-ray photoelectron spectroscopy (XPS) was applied to analyze the surface composition via an ESCALAB 250 device. Raman analysis was performed via a Renishaw inVia microprobe. The magnetic measurements were conducted at 300 K via a commercial Physical Property Measurement System (PPMS-9) (Quantum Design, United States) equipped with a vibrating sample magnetometer (VSM). The T_2_-weighted MRI was recorded via an MRI device (T_2_) by using a 1.5 T SIGNA Voyager MRI device. The light source at 808 nm was derived from a near-infrared radiation laser device (DBAL-I2). Infrared radiation images with various temperatures were recorded via an infrared radiation camera (FLIR T640-45) at regular time intervals.

### 2.4 Procedures for photothermal therapy

The cell culture medium used was DMEM medium, 10% fetal bovine serum albumin, and 1% penicillin–streptomycin double antibody solution. A 20 mg/L Au/Fe_3_O_4_@C NG suspension was added to the coverslips to incubate esophageal cancer cells for 3 h. Cells were cultured in a CO_2_ incubator at 37°C with a CO_2_ concentration of 5%. Newly prepared LIVE/DEAD solution obtained from Thermo Fisher Scientific was applied to rinse the cells several times. Then, laser radiation (wavelength 808 nm, power density 0–2.0 W/cm^2^) was applied to radiate the esophageal cancer cells. The cell images were recorded on a ZEISS Axiocam microscope camera.

### 2.5 Magnetic hyperthermia experiments

A homogeneous alternating current magnetic field with a frequency of 300 kHz and an amplitude of 200–800 Oe was applied for the magnetic hyperthermia of Au/Fe_3_O_4_@C NGs via a calorimetric approach. In addition, photothermal experiments induced by laser radiation (808 nm of wavelength, 0–2.0 W/cm^2^ of power densities) were carried out in combination with magnetic hyperthermia.

## 3 Results and discussion

### 3.1 Characterization of Au/Fe_3_O_4_@C NGs

**FIGURE 1:**
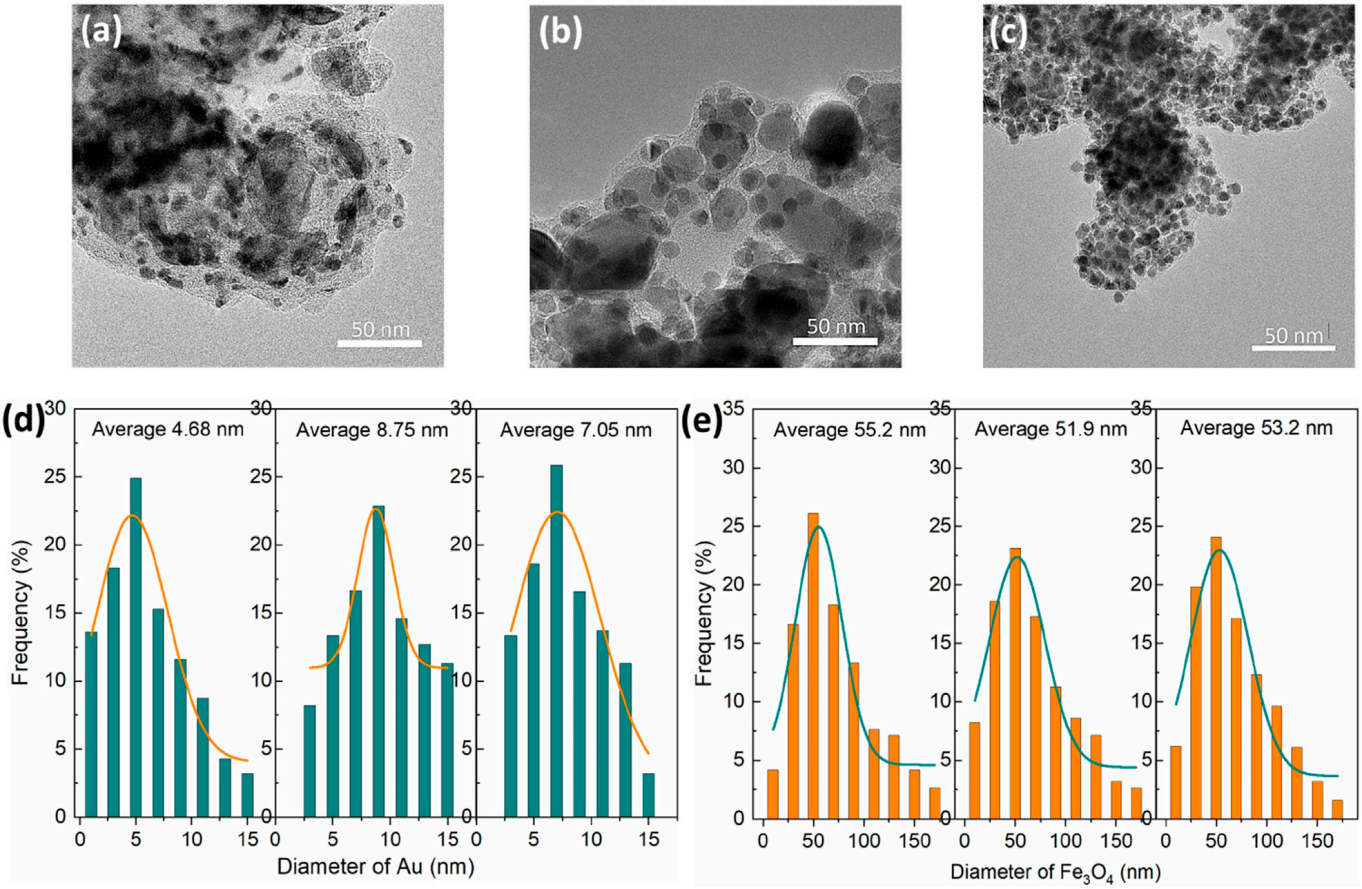
TEM images of Au/Fe_3_O_4_@C-1 to -3 NGs **(A–C)**. Diameters of Au **(D)** and Fe_3_O_4_ **(E)** in Au/Fe_3_O_4_@C NGs.

Au/Fe_3_O_4_@C NGs prepared with a mixed gas of C_2_H_4_/O_2_ (4.0 L/min with three specific ratios including 3.9/0.1 L/min, 3.5/0.5  L/min, and 3.0/1.0 L/min) were termed as Au/Fe_3_O_4_@C-1 to -3 NGs. Au/Fe_3_O_4_@C NGs were composed of Au NPs and Fe_3_O_4_ NPs separately coated with carbon shells (Figures 1A–C). Fe_3_O_4_ core sizes changed from 51.9 nm to 55.2 nm (Figures 1D, E). The PDI values for Au/Fe_3_O_4_@C-1 NGs, Au/Fe_3_O_4_@C-2 NGs, and Au/Fe_3_O_4_@C-3 NGs were 0.572, 0.593, and 0586, respectively. The thickness of the carbon shell to coat the Fe_3_O_4_ core decreased from Au/Fe_3_O_4_@C-1 to Au/Fe_3_O_4_@C-3. Carbon-encapsulated Au NPs with a shell thickness of 2–4 graphite layers were located and attached with carbon-encapsulated Fe_3_O_4_ NPs. Au core sizes changed from 4.68 nm to 8.75 nm. Carbon coating shells with 2–4 layers would exert a slight shielding effect toward Au cores. Namely, the plasmonic property of carbon-encapsulated Au NPs should be dominated by Au cores, which may be favorable for the photothermal performance of Au/Fe_3_O_4_@C NGs.

The formation process of Au/Fe_3_O_4_@C NGs follows: When the enclosed flame spray pyrolysis started, the precursors, including gold acetate, ferric acetate, and carbon source C_2_H_4,_ would be decomposed by the enclosed flame spray (Li et al., 2017; Mostafa et al., 2021). The species of Au and Fe would coalesce to form NGs as Fe_3_O_4_/Fe_3_C-Au owing to their collision. The carbon species would react with these nanoclusters as a dissolution in Fe NPs and a deposition on the surface of Au NPs (low solubility for carbon). Considering the high temperature of enclosed flame spray, carbon deposited on the surface of Au NPs would form graphite coating shells over Au NPs because of the catalysis of Au for carbon. Graphite coating shells over Fe_3_O_4_ NPs would also be formed by the re-precipitated carbon from the Fe-based cores. Finally, Au/Fe_3_O_4_@C NGs would be formed.

**FIGURE 2:**
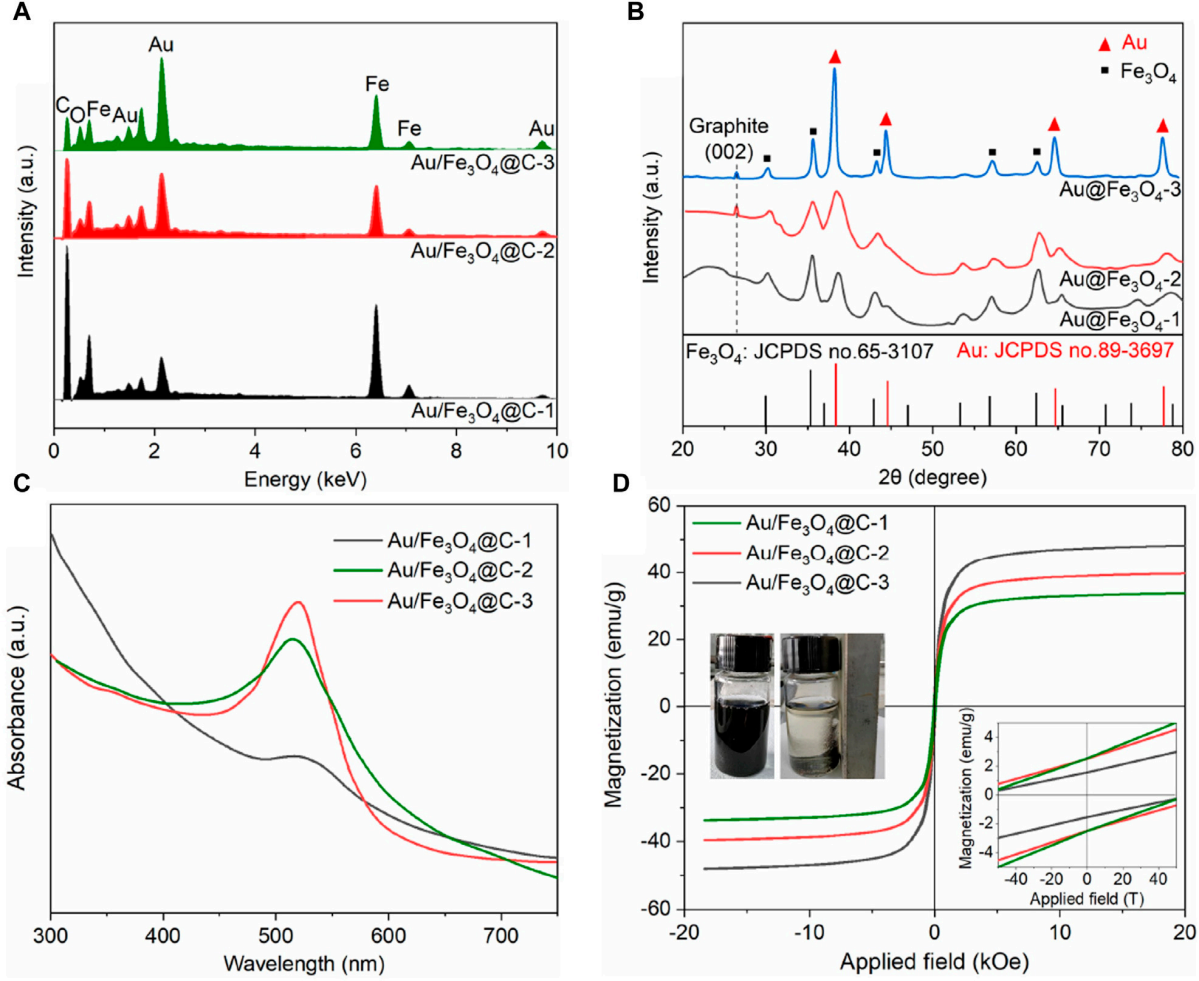
EDS spectra **(A)**, XRD **(B)**, UV/Vis/NIR absorbance **(C),** and magnetic hysteresis loops **(D)**. A separation image of Au/Fe_3_O_4_@C NGs in aqueous solutions with an applied magnetic field was inserted.

EDS spectra (Figure 2A) confirmed that the typical elemental compositions of Au/Fe_3_O_4_@C NGs were Au, Fe, C, and O without any other impurities. The Au content was 24.4 wt% for Au/Fe_3_O_4_@C-1, 33.3 wt% for Au/Fe_3_O_4_@C-2, and 42.8 wt% for Au/Fe_3_O_4_@C-3, while those of Fe element were 31.3 wt% for Au/Fe_3_O_4_@C-1, 29.7 wt% for Au/Fe_3_O_4_@C-2, and 23.9 wt% for Au/Fe_3_O_4_@C-3. The crystalline structures of Au/Fe_3_O_4_@C NGs analyzed by XRD are shown in Figure 2B. A diffraction peak located at 26.5° was derived from the (002) crystalline reflection of graphite. Characteristic peaks at 38.5° ((111) reflection) and 44.7° ((200) reflection) demonstrated the existence of Au (JCPDS no. 89-3697) in Au/Fe_3_O_4_@C NGs (Chang et al., 2021). Meanwhile, peaks ascribed to Fe_3_O_4_ (JCPDS no.65-3107) were also observed, which verified that the Au/Fe_3_O_4_@C NGs were composed of three components, including carbon, Au, and Fe_3_O_4_ (Wang Z. et al., 2021). Given the localized surface plasmon resonance property of nanosized Au in Au/Fe_3_O_4_@C NGs, characteristic absorption bands were displayed for Au/Fe_3_O_4_@C-1 at 512 nm, for Au/Fe_3_O_4_@C-2 at 515 nm, and for Au/Fe_3_O_4_@C-3 at 519 nm (Figure 2C). With the increasing carbon component from 13.2 wt% to 32.3 wt%, the localized surface plasmon resonance induced by plasmonic coupling was broadened, and the absorption intensity was reduced. These broad optical absorption peaks were located in the visible and infrared regions, which allowed us to assess their applicability in photothermal therapy. As shown by the magnetic hysteresis loops of Au/Fe_3_O_4_@C NGs displayed in Figure 2D, the values of saturation magnetization of Au/Fe_3_O_4_@C NGs rose gradually, that is, 33.7 emu/g (58.6 Oe of coercivity), 39.7 emu/g (62.9 Oe of coercivity), and 48.2 emu/g (75.4 Oe of coercivity) for Au/Fe_3_O_4_@C-1 to -3 NGs, separately. Enclosed flame spray pyrolyzed multicomponent Au/Fe_3_O_4_@C NGs showed weak ferromagnetism because of the presence of the Fe domain in the NGs (Li et al., 2021; Li et al., 2022). The zeta potentials of Au/Fe_3_O_4_@C-1 NGs, Au/Fe_3_O_4_@C-2 NGs, and Au/Fe_3_O_4_@C-3 NGs at pH 5.0–7.0 were measured, as shown in Supplementary Figure S2 in the Supplementary Material. For instance, the zeta potentials of Au/Fe_3_O_4_@C-1 NGs, Au/Fe_3_O_4_@C-2 NGs, and Au/Fe_3_O_4_@C-3 NGs at pH 7.0 were −0.22, −0.34, and −0.19, respectively. Moreover, the EDS mapping of the nanoformulation image has been provided, as shown in Supplementary Figure S3, to reveal the actual distribution of elements in the nanoformulations.

To analyze stability, 100 mg/L of Au/Fe_3_O_4_@C-3 NGs was obtained from 100 μg of Au/Fe_3_O_4_@C-3 NGs resuspended in 1.0 mL of deionized water, and the absorption was further measured at pre-set time points (0 h, 12 h, 24 h, and 36 h). As shown in Supplementary Figure S4A, the characteristic absorption spectra of Au/Fe_3_O_4_@C-3 NGs at 517 nm displayed a negligible change over this time period. There was little change in the size of Au/Fe_3_O_4_@C-3 NGs after this time period (Supplementary Figure S4B).

### 3.2 MRI study

**FIGURE 3:**
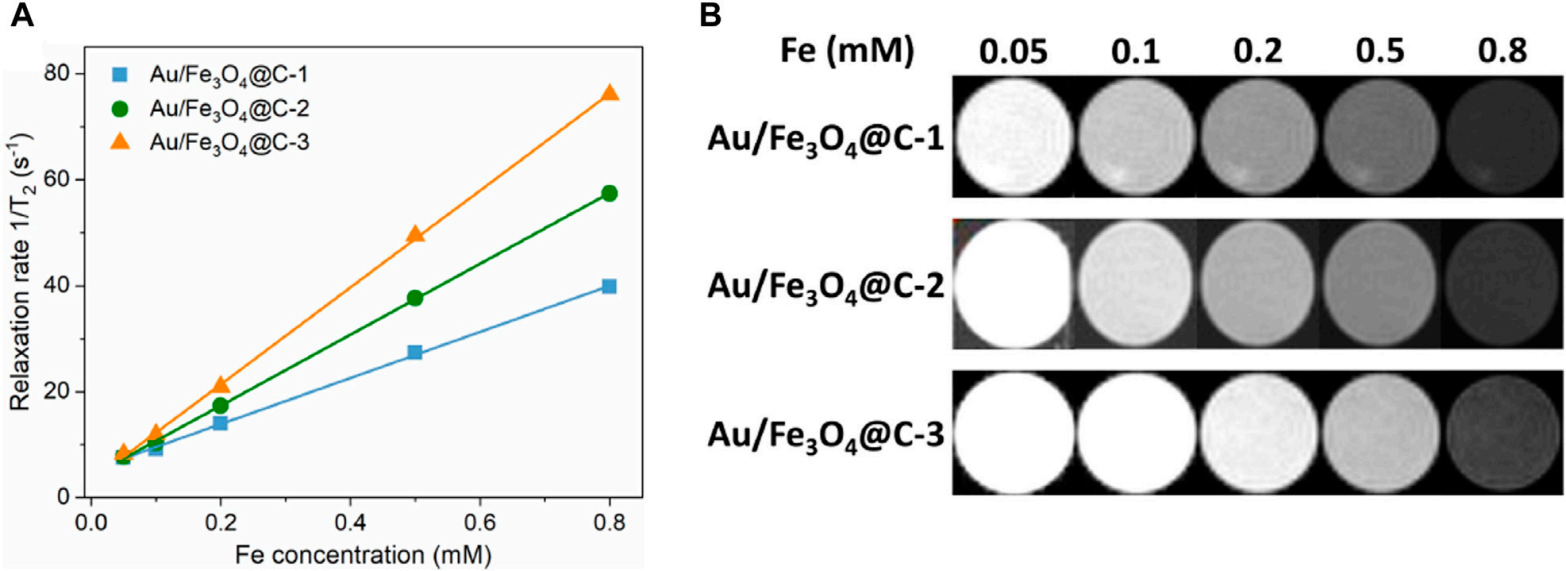
Effect of Fe concentration in Au/Fe_3_O_4_@C NGs on the inverse transverse relaxation time (1/T_2_) **(A)** and T_2_-weighted MRI **(B)**.

The application of MRI agents based on iodine is restricted because of their relatively low X-ray absorption coefficient. Magnetic materials containing an Fe component have presented superior MRI performance and can be applied as a computerized tomography (CT) contrast agent. Figure 3A showed the effect of Fe concentration in Au/Fe_3_O_4_@C NGs on the transverse proton relaxation time (1/T_2_) of CT signals, where the Fe concentration was measured via an inductively coupled plasma atomic emission spectrometry method. Relaxation time 1/T_2_ was linearly correlated with increasing Fe concentration in Au/Fe_3_O_4_@C NGs. Specifically, as the concentration of Fe increased from 0.05 mM to 0.8 mM, the values of 1/T_2_ increased from 7.2 s^−1^ to 39.7 s^−1^, 57.4 s^−1^, and 76.0 s^−1^ for Au/Fe_3_O_4_@C-1 to -3 NGs, separately. Figure 3B shows CT images of Au/Fe_3_O_4_@C NGs as a function of Fe concentration in Au/Fe_3_O_4_@C NGs. The values of measured relaxivity (r_2_) were 43.5 mM^−1^ s^−1^, 66.9 mM^−1^ s^−1^ and 91.4 mM^−1^ s^−1^ for Au/Fe_3_O_4_@C-1 to -3 NGs separately. The MRIs of Au/Fe_3_O_4_@C NGs tended to darken as the concentration of Fe increased from 0.05 mM to 0.8 mM. Considering these MRI results, Au/Fe_3_O_4_@C NGs could be applied as an excellent agent for the potential CT application.

### 3.3 Photothermal performance study

**FIGURE 4:**
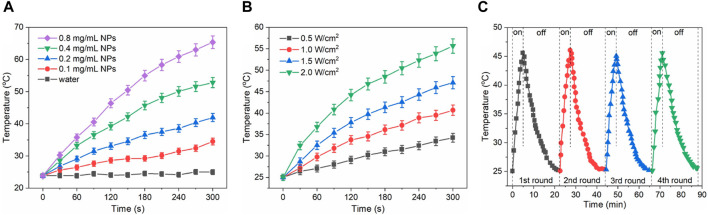
Effect of Au/Fe_3_O_4_@C-3 concentration on the photothermal heating property of solutions **(A)**. Effect of power densities on the property of solutions of solutions **(B)**. Stability evaluation of photothermal property **(C)**.

Given the efficient absorbance of Au/Fe_3_O_4_@C-3 NGs in the region of UV-Vis to NIR, the effect of Au/Fe_3_O_4_@C-3 concentration from 0 mg/mL to 0.8 mg/mL on the photothermal performance of Au/Fe_3_O_4_@C-3 solutions was investigated with laser radiation (wavelength 808 nm, power density 1.0 W/cm^2^, and duration 300 s). An obvious concentration-dependent temperature enhancement was observed for the aqueous suspensions of Au/Fe_3_O_4_@C-3 NGs (Figure 4A). The temperatures of 0.1 mg/mL, 0.2 mg/mL, 0.4 mg/mL, and 0.8 mg/mL of Au/Fe_3_O_4_@C-3 NGs suspensions reached 34.5°C, 41.9°C, 52.8°C, and 65.4°C, respectively, after only 300 s irradiation. The effect of power density (0.5–2.0 W/cm^2^) on the photothermal performance of Au/Fe_3_O_4_@C-3 solutions (0.2 mg/mL) is shown in Figure 4B. Distinct laser-power-dependent photothermal behaviors of Au/Fe_3_O_4_@C-3 suspensions are displayed; that is, the temperatures of Au/Fe_3_O_4_@C-3 suspensions reach 34.3°C, 40.7°C, 47.1°C, and 55.6°C with the increased power densities after only 300 s of laser irradiation. The thermal energy converted from the laser was positively related to both the concentration of Au/Fe_3_O_4_@C-3 NGs and the power densities of the laser. Moreover, the stability of this conversion by Au/Fe_3_O_4_@C-3 NGs was evaluated for four cycles with a laser radiation duration of 300 s (Figure 4C). The amount of temperature increase of the aqueous suspension changed only slightly, indicating that the stability of photothermal conversion by Au/Fe_3_O_4_@C-3 NGs was excellent.

### 3.4 Photothermal tumor-ablation study

**FIGURE 5:**
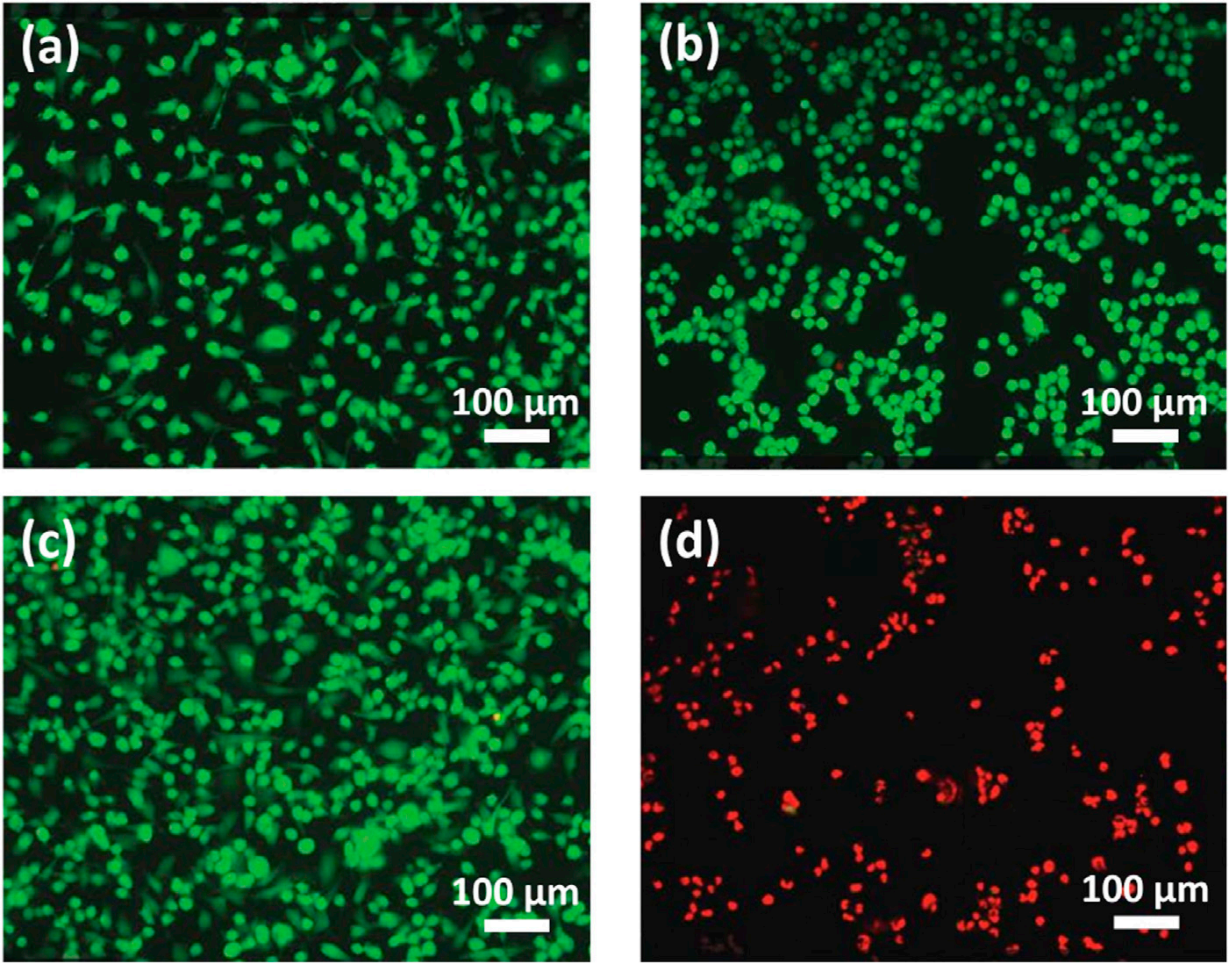
Fluorescent images (no laser radiation) of cells **(A)** and cells with Au/Fe_3_O_4_@C-3 NGs (50 mg/L) **(B)**. Fluorescent images (with laser radiation) of cells **(C)** and cells with Au/Fe_3_O_4_@C-3 NGs (50 mg/L) **(D)**.

Enclosed flame spray pyrolyzed Au/Fe_3_O_4_@C-3 NGs were applied as a photothermal tumor-ablation agent to a mixture of esophageal cancer cells and Au/Fe_3_O_4_@C-3 NGs. The conditions for the laser radiation are wavelength 808 nm, power density 2.0 W/cm^2^, duration 5.0 min, and spot size 5 mm. The fluorescent images of cell viability shown in Figure 5 indicate that green was utilized to mark the cell, while red color was used to mark dead cells. The temperature of esophageal cancer cells in the absence of laser radiation was 19.3°C (Figure 5A) as a comparison. The esophageal cancer cells incubated with Au/Fe_3_O_4_@C-3 NGs (50 mg/L) without any laser irradiation and the esophageal cancer cells alone with laser irradiation are shown in Figures 5B, C; the temperatures reached 20.6°C and 21.4°C, respectively. The viability of esophageal cancer cells remained stable with the addition of Au/Fe_3_O_4_@C-3 NGs in the absence of laser irradiation, indicating the good biocompatibility and weak toxicity of Au/Fe_3_O_4_@C-3 NGs. Meanwhile, the temperature of esophageal cancer cells without the addition of Au/Fe_3_O_4_@C-3 NGs increased lightly due to the laser radiation but presented no obvious cell damage. The above phenomena indicate that such treatments do not compromise cell viability (Cerezo-Navarrete et al., 2022; Chu et al., 2022).

In contrast, the slide temperature in the presence of Au/Fe_3_O_4_@C-3 NGs rose to 70.3°C (Figure 5D), inducing a significant killing of esophageal cancer cells by the laser. It is demonstrated that the enclosed flame spray pyrolyzed Au/Fe_3_O_4_@C-3 NGs could be an efficient photothermal agent to produce significant environmental energy from the laser, which could kill the esophageal cancer cells without obvious impact on the healthy tissues. *In vitro* particle uptake experiments were carried out. Au/Fe_3_O_4_@C-3 NGs were incubated with esophageal cancer cells for 24 h at an extracellular iron concentration of 100 × 10^−6^ mol/L (Table 1). The 24 h incubation at 100 × 10^−6^ mol/L of Fe resulted in a mass of iron uptaken by cells of 3.0 ± 0.4 pg of Fe per cell.

**TABLE 1:** Uptake of Au/Fe_3_O_4_@C-3 NGs in cells at an extracellular Fe concentration of 100 × 10^−6^ mol/L for different incubation times.

Incubation Fe concentration	Incubation time	Fe mass [pg_Fe_/cell]
0.1	24	3.0 ± 0.4
0.1	3	0.51 ± 0.08
0.05	3	0.31 ± 0.06
0.025	3	0.16 ± 0.09

### 3.5 Magnetic hyperthermia study

**FIGURE 6:**
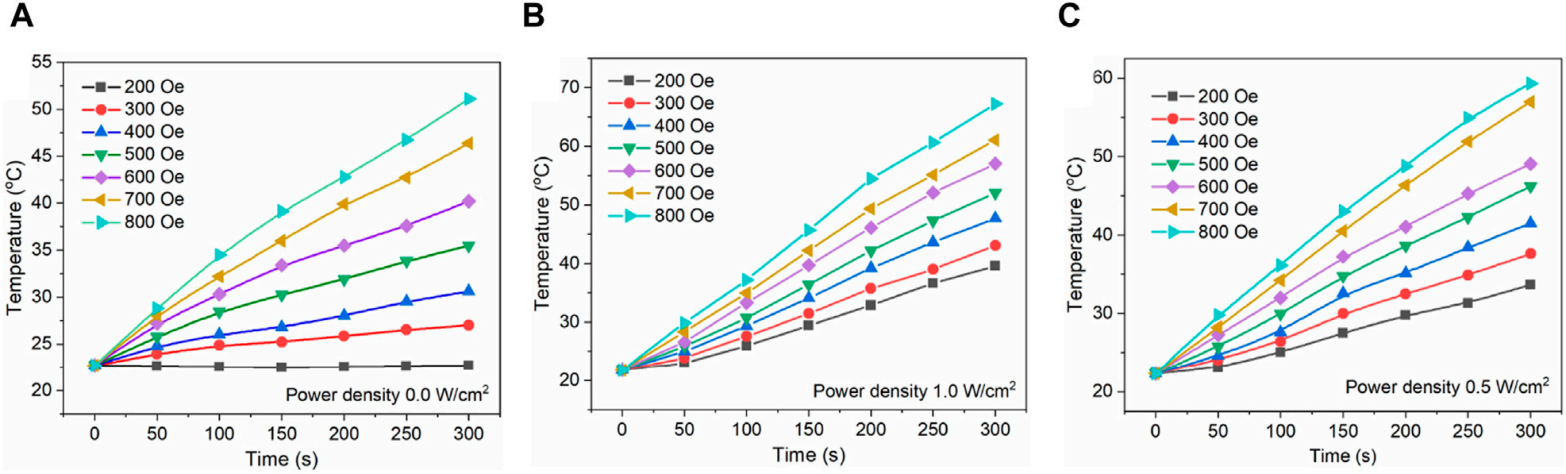
Magnetic hyperthermia for Au/Fe_3_O_4_@C-3 NGs under a magnetic field of 200–800 Oe and 300 kHz **(A)**. Heating curves for Au/Fe_3_O_4_@C-3 NGs under a magnetic field of 200–800 Oe and a laser irradiation of 0.5 W/cm^2^ **(B)** and 1.0 W/cm^2^ **(C)**.

To restrain the physical motion of Au/Fe_3_O_4_@C-3 NGs, Au/Fe_3_O_4_@C-3 was added into agar with a mass concentration of 2.0 wt%. The temperatures of magnetic hyperthermia with different external alternating current applied magnetic fields were measured as shown in Figure 6A. The heating temperature increases with the applied magnetic field, that is, 33.7°C for 200 Oe, 37.6°C for 300 Oe, 41.5°C for 400 Oe, 46.2°C for 500 Oe, 49.1°C for 600 Oe, 56.9°C for 700 Oe, and 59.3°C for 800 Oe. Because the therapeutic temperature window is located at temperatures ranging from 40°C to 44°C, which enable damaging cancer cells while preserving healthy cells, a magnetic field >400 Oe should be applied in magneto-calorific experiments.

Moreover, the magnetic field dose should be kept as low as possible in the practical application considering its negative impact on normal cells (Mai et al., 2022; Delgado et al., 2023; Myrovali et al., 2023; Wang et al., 2023). Because both the photothermal temperature and magnetic-heating temperature of Au/Fe_3_O_4_@C NGs were positively related to the laser power density and magnetic field value separately, it is contradictory to improve the efficiencies of photothermal therapy and magnetic hyperthermia without introducing injury to the cell tissues. Given that, we applied the laser radiation and magnetic field simultaneously to investigate the heating performance of Au/Fe_3_O_4_@C NGs. As the heating curves shown in Figures 6B, C, the temperatures under combined external conditions are obviously higher than those obtained without laser irradiation. For instance, the obtained temperature after 5 min under the magnetic field of 400 Oe increases from 30.6°C to 41.5°C and 47.4°C with the combination of laser exposure (power density: 0.5 W/cm^2^ and 1.0 W/cm^2^ separately). Without applying the laser irradiation, the required value for the magnetic field to enable the target temperature of 40°C was 600 Oe. After adding laser exposure (power density: 0.5 W/cm^2^ and 1.0 W/cm^2^ separately), the required magnetic field value decreases to 400 and 300 Oe, respectively. Thus, it is believed that the usage of these two external stimuli showed a higher heating efficiency than the situation under separate stimuli. The above findings demonstrated that Au/Fe_3_O_4_@C NGs combined with plasmonic and magnetic components could be promising hyperthermia agents with reduced required laser radiation and magnetic field doses. Furthermore, carbon coating shells over Au/Fe_3_O_4_ NGs would present a favorable biocompatibility because carbon materials are generally tolerated by organisms.

## 4 Conclusion

In conclusion, one-step enclosed flame spray pyrolyzed Au/Fe_3_O_4_@C NGs were tailored; that is, Fe_3_O_4_ cores (51.9–55.2 nm) were coated with a decreasing carbon shell thickness, and Au cores (4.68–8.75 nm) were coated with 2–4 layered graphite shells by tuning the carbon shell thickness through changing C_2_H_4_ content in the reacting gas mixture. The saturation magnetization was enhanced from 33.7 emu/g to 48.2 emu/g, while the optical absorption of Au/Fe_3_O_4_@C NGs was also significantly aroused. The carbon shell allowed the good dispersion of NGs without laser-induced and magnetic field-induced merging of Au/Fe_3_O_4_. Under 5 min of laser irradiation at 808 nm, excellent MRI properties (43.5–91.4 mM^−1^ s^−1^) and photothermal performances (34.5–65.4°C with the addition of 0.1–0.8 mg/mL of Au/Fe_3_O_4_@C-3 NGs, and 34.3-55.6°C with the power density of 0.5–2.0 W/cm^2^) were presented, showing an efficient killing of esophageal cancer cells. Moreover, the combined application of photothermal and magnetic heating properties of Au/Fe_3_O_4_@C NGs reduced the required intensity of both the laser and the magnetic field compared to the intensity of separate situations. Compared with other findings, the preparation approach of Au/Fe_3_O_4_@C NGs was facile and easily controlled. Furthermore, the carbon coating shell enabled the improved stability and biocompatibility of the Au/Fe_3_O_4_@C agents. This study provided meaningful insights into integrating magnetic and plasmonic components with carbon coating shells for chemo-photothermal synergistic cancer therapy.

## Data Availability

The original contributions presented in the study are included in the article/Supplementary Material; further inquiries can be directed to the corresponding authors.
