# Supplementary material for: Flame spray pyrolyzed carbon-encapsulated Au/Fe3O4 nanoaggregates enabled efficient photothermal therapy and magnetic hyperthermia of esophageal cancer cells
Source: Front Bioeng Biotechnol. 2024 May 28;12:1400765. doi: 10.3389/fbioe.2024.1400765 (PMC11165064; doi:10.3389/fbioe.2024.1400765)
Supplement: Supplementary file 1 [file DataSheet1.docx]

Flame spray pyrolyzed carbon-encapsulated Au/Fe_3_O_4_ nanoaggregates enabled efficient photothermal therapy and magnetic hyperthermia of esophagus cancer cells

Zida Wang,^1,†^ Gongzhe Liu,^2,†^ Jiangping Zhou,^3,†^ Xiaogang Zhao,^4,*^ Jie Cai^4,^^[[1]](#footnote-1)^*

^1^Department of Emergency, Shanghai Pulmonary Hospital, School of Medicine, Tongji University, Shanghai 200433, China.

^2^Department of Cardiothoracic Surgery, People's Hospital Affiliated to Shandong First Medical University, Jinan 271199, China

^3^Department of Anesthesiology, Shanghai Pulmonary Hospital, School of Medicine, Tongji University, Shanghai 200433, China.

^4^Department of Thoracic Surgery, Shanghai Pulmonary Hospital, School of Medicine, Tongji University, Shanghai 200433, China.


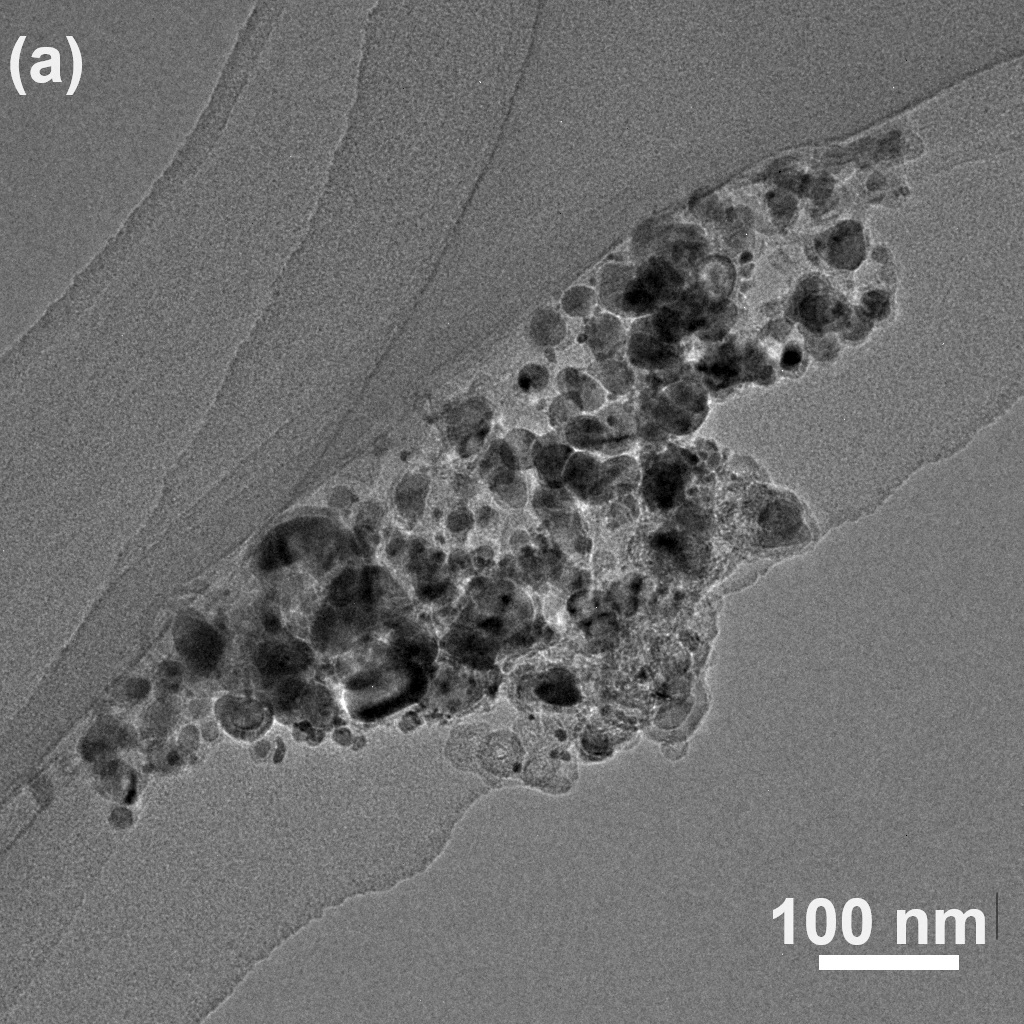

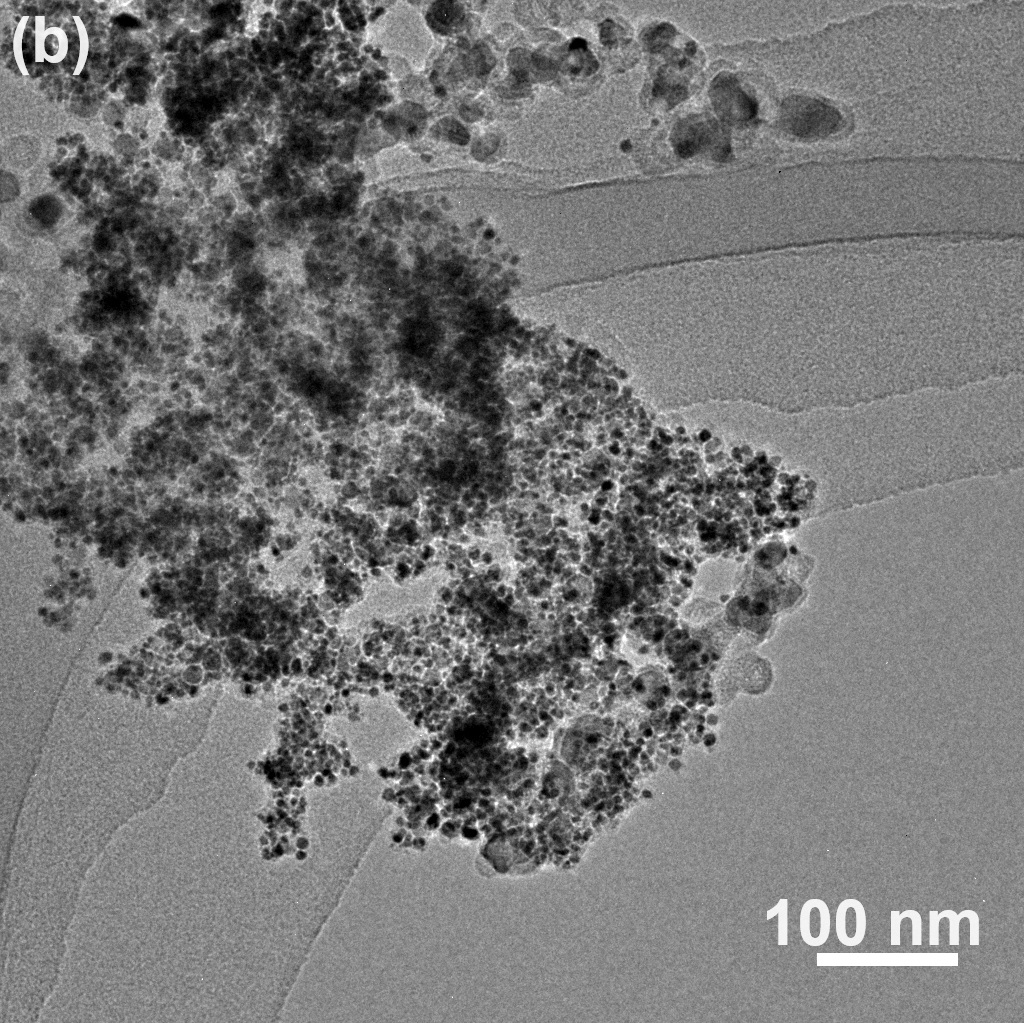

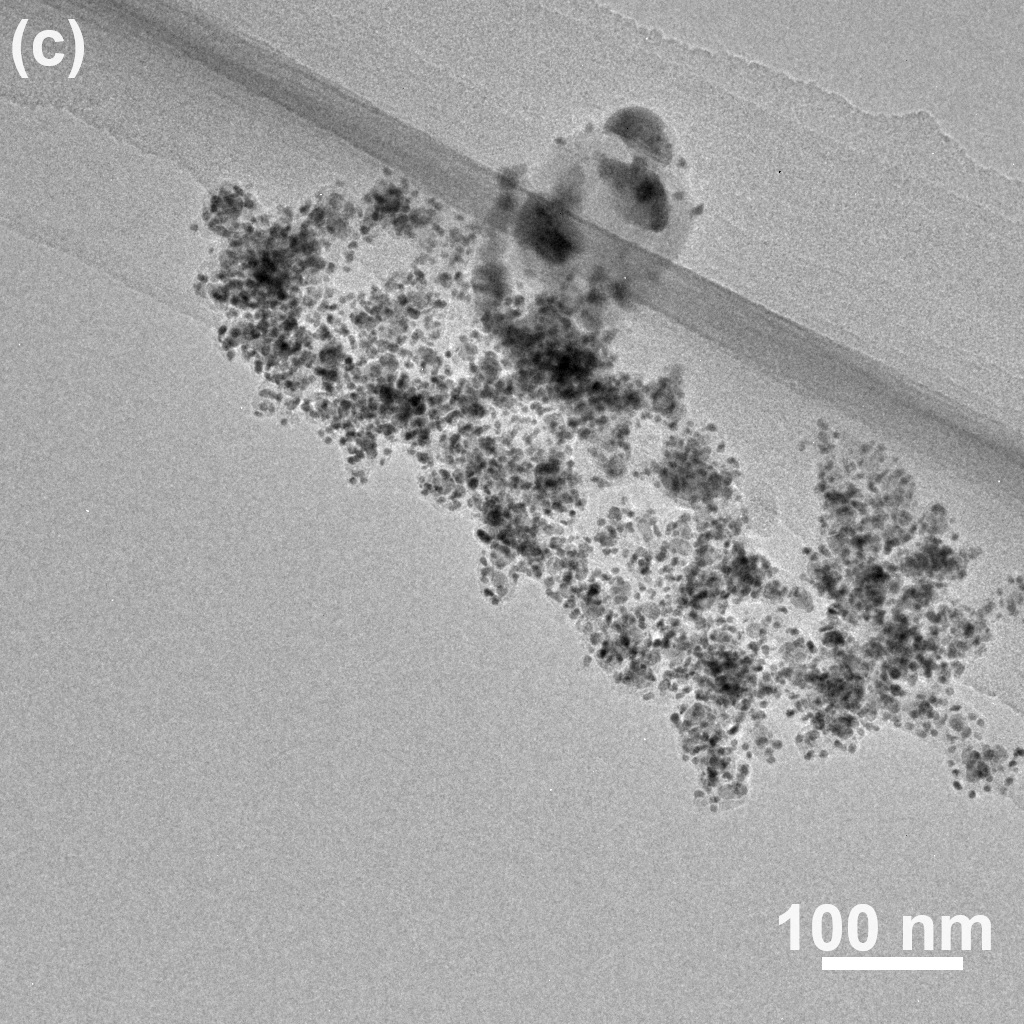


Figure S1. TEM images of Au/Fe_3_O_4_@C-1 (a), Au/Fe_3_O_4_@C-2 (b), and Au/Fe_3_O_4_@C-3 (c).





Fig. S2. Zeta potentials of Au/Fe_3_O_4_@C-1 NGs, Au/Fe_3_O_4_@C-2 NGs and Au/Fe_3_O_4_@C-3 NGs at pH 5.0-7.0.


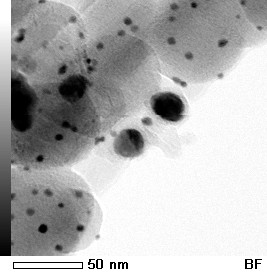

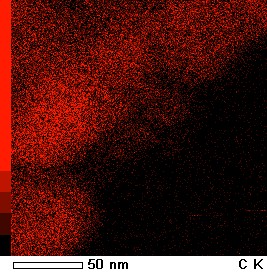


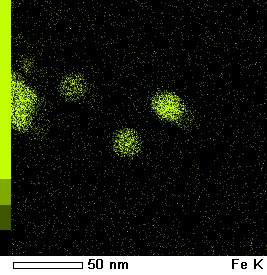

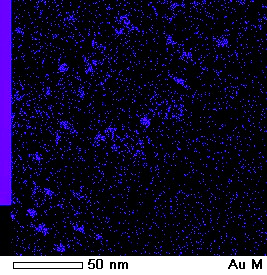


Fig. S3. EDS mapping of Au/Fe_3_O_4_@C-3 NGs.







Fig. S4. The stability of Au/Fe_3_O_4_@C-3 NGs. UV-vis absorption spectra peak stability (a) and hydrodynamic size distributions (b) at 0, 12 and 36 hours.

1. ^†^These authors contributed equally to this work.

   *Corresponding author. *Email addresses*: cj_shfk@163.com, and zxg_shfk@126.com [↑](#footnote-ref-1)
